# Development and Performance Evaluation of T-prep24: A Novel Automated Nucleic Acid Extraction System Based on Silica Magnetic Beads

**DOI:** 10.3390/diagnostics15121528

**Published:** 2025-06-16

**Authors:** Jung Ho Park, Naeun Kwak, Dokyun Kim, Jong-Chan Chae, Seok Hoon Jeong

**Affiliations:** 1 Division of Biotechnology, Jeonbuk National University, Iksan 54596, Republic of Korea; 2 BioPark Diagnostics Inc., Seoul 01809, Republic of Korea; 3 Department of Laboratory Medicine, Research Institute of Bacterial Resistance, Yonsei University College of Medicine, Seoul 06273, Republic of Korea; 4 Advanced Institute of Environment and Bioscience, Jeonbuk National University, Iksan 54596, Republic of Korea

**Keywords:** nucleic acid extraction, SARS-CoV-2, PCR

## Abstract

**Background:** Rapid molecular detection of infectious pathogen with high sensitivity and specificity has become increasingly important in clinical microbiology laboratories. The need to develop domestically produced nucleic acid extraction equipment has grown since COVID-19 pandemic in South Korea. In this study, we developed a new magnetic bead-based automated nucleic acid extraction system, T-Prep24 system, and the performance of the new system was evaluated with many clinical specimens. **Methods:** A total of 180 respiratory specimens were collected, and nucleic acids were extracted using three different systems, the T-Prep24 system, TANBead system, and Qiagen system. The quality and concentration of extracted nucleic acid were evaluated by spectrophotometer and Qubit fluorometer. Qualitative determination for SARS-CoV-2 was performed by PowerChek SARS-CoV-2 Real-time PCR kit. **Results:** The median concentration of nucleic acid extracted by T-Prep24 system and measured by a fluorescence-based method was 0.685 ng/µL (first to third interquartile range, 0.258–1.493 ng/µL), which was lower than that of nucleic acid extracted by TANBead system (median value, 0.985 ng/µL; first to third interquartile range, 0.610–1.583 ng/µL; *p* < 0.001), and that of nucleic acid extracted by Qiagen system (median value, 4.710 ng/µL; first to third interquartile range, 3.783–5.810 ng/µL; *p* < 0.001). The Cq values of PCR assays using nucleic acid extracted by T-prep24 showed minimal systematic bias (slope = 1.015) when compared with those using nucleic acid extracted by TANBead, but significant proportional constant bias (slope = 0.907) when compared with those using nucleic acid extracted by Qiagen. The results of PCR assays using nucleic acid extracted by the T-Prep24 system were identical to those of PCR assays using nucleic acid extracted by TANBead system, and two discrepant results were identified when comparing with those by the Qiagen system. **Conclusions:** T-Prep24 system is a reliable and effective tool for nucleic acid extraction in clinical settings. Future investigations should be carried out to widen the applicability to a range of pathogens and sample types.

## 1. Introduction

Rapid and accurate molecular detection of infectious pathogen with high sensitivity and specificity has become crucial in clinical microbiology laboratories, particularly due to increasing demand for timely diagnosis and treatment decision. The need for high sensitivity and specificity in detecting a wide range of infectious organisms, including bacteria and viruses, is raised by the importance of diagnostic stewardship. Advances in the molecular techniques, including polymerase chain reaction (PCR), real-time PCR, and nucleotide sequencing, have significantly shortened the test turnaround time for laboratory testing, and enhanced the overall accuracy of pathogen detection [1].

Nucleic acid extraction is the first step of molecular tests, and the extracted nucleic acid by adequate process and equipment should be used for a proper examination. Nucleic acid extraction is composed of four steps—cell disruption, removal of membrane lipids and proteins, nucleic acid purification, and nucleic acid concentration [2,3]. The initial and critical step in this process is cell disruption. Depending on the type of specimen and target organisms, various methods could be employed. Chemical methods include osmotic shock, enzymatic digestion, the use of detergent, and alkali treatment, while mechanical methods include homogenization, ultrasonication, and application of pressure. A proper cell disruption method greatly influences the yield and quality of extracted nucleic acids.

For the molecular detection of virus infection with clinical specimens, nucleic acid extraction should be performed with effective separation, avoidance of contamination, and avoidance of interference with other substances contained in the specimen. Manual nucleic acid extraction methods have been widely used in the laboratories [4]; however, the methods were labor-intensive and prone to contamination, potentially resulting in false positive results. Recently, many automated extraction systems have been introduced to clinical laboratories for routine diagnostics such as QIAsymphony and TANBead Smart Lab Assist system [5,6]. These automated devices have been widely adopted due to the standardized extraction procedure and reduction in hands-on time and human error, and they have been reported to show robust performance both for multiple bacterial and viral detection panels based on multiplex real-time PCR and for high-throughput microbial community profiling based on next-generation sequencing [7]. In addition, the need to develop domestically produced nucleic acid extraction equipment has grown since COVID-19 pandemic in South Korea.

**Figure 1:**
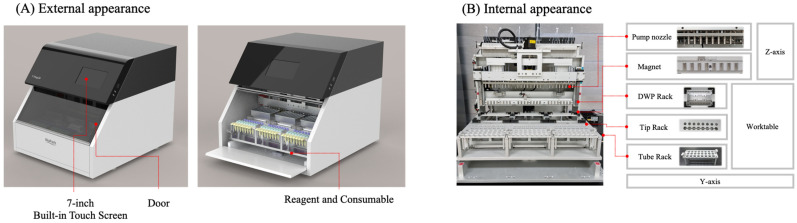
External and internal appearances of T-Prep24 system. (**A**) External apprearance. (**B**) Internal appearance.

**Figure 2:**
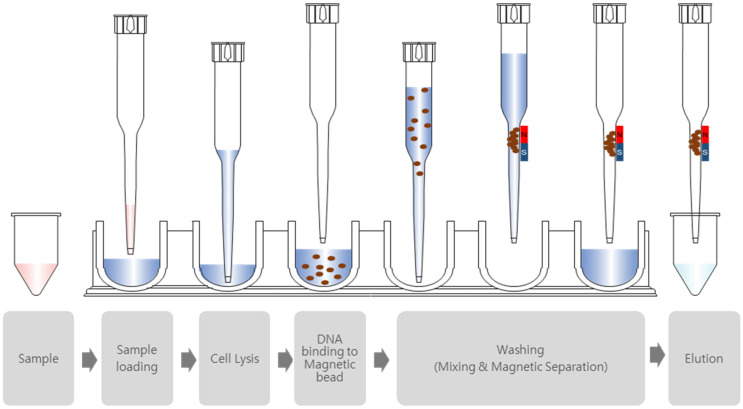
Operation process of T-Prep24 nucleic acid extraction system. The process of nucleic acid extraction in this automated system is as follows: (1) sample loading, (2) cell lysis, (3) nucleic acid adsorption to magnetic bead, (4) washing, and (5) elution.

T-Prep24 (BioPark Diagnostics Inc., Seoul, Republic of Korea), automated nucleic acid extraction system based on silica magnetic bead method, has recently been developed. The T-prep24 system consists of a syringe unit, magnetic unit, plate rack for reagents, tip rack for tips and piercers, and tube rack for samples and additional reagents (Figure 1). The process of nucleic acid of this automated system is as follows: (1) sample loading, (2) cell lysis, (3) nucleic acid adsorption to magnetic bead, (4) first magnetic separation, (5) washing, (6) second magnetic separation, and (7) elution (Figure 2). Three types of buffers, including lysis and binding buffer, and two types of washing buffers were developed. Lysis and binding buffer of the T-prep24 system were developed with guanidine thiocyanate, chaotropic agent, and proper salt. The concentration of proper salt component was optimized for the washing buffer 1, and washing buffer 2 was developed with optimal concentration of ethanol for the removal of contaminants and rapid drying process. A total of 24 samples could be loaded in each run, and processing time for one run is about 30 min. The T-prep24 system has advantages in minimizing aerosol contamination due to its closed system, disposable pipet tip, and ultraviolet lamp, and it showed high extraction efficiency with heating block in lysis and elution wells. This system has advantages in preventing contamination by its closed system, ultraviolet decontamination process, and narrow pipet design.

T-Prep24 is designed for clinical specimens including whole blood, plasma, swabs, and stools; however, the performance of this system has not been evaluated. In this study, the performance of nucleic acid extraction of T-Prep24 has been evaluated by performing SARS-CoV-2 PCR assay.

## 2. Materials and Methods

### 2.1. Clinical Samples

A total of 180 respiratory samples including 70 positive samples and 110 negative samples were included in this study. Nasopharyngeal and oropharyngeal swabs were collected and transported via virus transport media (AB Transport Medium & Swabs; AB MEDICAL, Seoul, Republic of Korea) from the patients undergoing evaluation of suspected SARS-CoV-2 infection.

### 2.2. Nucleic Acid Extraction

The nucleic acid of clinical samples was extracted by three nucleic acid extraction kits, including BPDX-Viral DNA/RNA Extraction Kit with the T-Prep24 equipment (T-Prep24 System), TANBead Nucleic acid extraction kit with SLA-E13200 (TANBead system; Taiwan Advanced Nanotech Inc., Taoyuan City, Taiwan), and QIAamp DSP Viral RNA Mini Kit with QIAcube (Qiagen system; Qiagen, Hilden, Germany) following the manufacturers’ instruction. The specification of three extraction systems is summarized in Table 1.

### 2.3. Measurement of Concentration and Quality of Extracted Nucleic Acid

The concentration and purity of extracted nucleic acid was measured by absorbance at 230 nm, 260 nm, and 280 nm using a spectrophotometer (NanoDrop Technologies, Wilmington, DE, USA). The absorbance was measured in triplicates and mean value was calculated. In addition, the concentrations of extracted nucleic acid were also determined using a Qubit Fluorometer (ThermoFisher Scientific, Wilmington, DE, USA) with RNA HS assay Kit (ThermoFisher Scientific).

### 2.4. Nucleic Acid Amplification Assay

Qualitative determination for SARS-CoV-2 was performed by PowerChek SARS-CoV-2 Real-time PCR kit following the manufacturers’ instruction. Five microliters of extracted nucleic acid was added to 15 μL of master mix. Recombinant RNA was included as an internal control to verify the validity of the amplification process. The real-time PCR was performed using CFX-96 Real Time System thermal cycler (Bio-rad, Hercules, CA, USA) with the following cycling conditions: 50 °C for 30 min, 95 °C for 10 min, 40 cycles of 95 °C for 15 s, and 60 °C for 1 min. A positive test result was defined as a Cq value under 38 in both *E* gene and *ORF1ab* gene.

### 2.5. Statistical Analysis

All statistical analyses in this study were performed using Analyse-it for Microsoft Excel version 5.30.5. The difference in concentration and purity indices of extracted nucleic acid by three different automated extraction systems were evaluated with Mann–Whitney U tests. The correlation of the Cq values of the *E* gene and *ORF1ab* genes with the results of positive samples tested with nucleic acid extracted by three different automated extraction systems were calculated by Passing-Bablok regression.

### 2.6. Ethical Statement

All processes in this study were conducted in accordance with the ethical standards of the institutional and national research committee, the 1964 Helsinki Declaration, and comparable ethical standards. The local institutional review board approved this study for the collection of residual nasopharyngeal and oropharyngeal swab samples and clinical data through a retrospective chart review (3-2022-0209).

## 3. Results

### 3.1. Concentration and Purity of Extracted Nucleic Acid According to the Nucleic Acid Extraction System

The median concentration of RNA extracted using the T-prep24 system measured by the Qubit RNA HS kit was 0.685 ng/µL (first to third interquartile range, 0.258–1.493 ng/µL), which was lower than that of extracted RNA by TANBead system (median value, 0.985 ng/µL; first to third interquartile range, 0.610–1.583 ng/µL), and that of RNA extracted by Qiagen system (median value, 4.710 ng/µL; first to third interquartile range, 3.783–5.810 ng/µL) (Table 2). The median concentration of nucleic acid extracted using the T-prep24 system measured by Nanodrop was 2.105 ng/µL (first to third interquartile range, 0.539–8.841 ng/µL), which was also lower than that by TANBead system (median value, 7.961 ng/µL; first to third interquartile range, 6.274–10.359 ng/µL, *p* < 0.001) and that by Qiagen system (median value, 83.275 ng/µL; first to third interquartile range, 74.434–87.454 ng/µL, *p* < 0.001). Median A260/A280 ratio measured by Nanodrop was 1.528, 1.511, and 2.652 with nucleic acid extracted using the T-prep24 system, TANBead system, and Qiagen system, respectively.

### 3.2. Result of SARS-CoV-2 PCR Assay

The result of SARS-CoV-2 PCR assay with nucleic acid extracted using the T-prep24 system, TANBead system, and Qiagen system are summarized in Table 2. Median Cq values of internal control of SARS-CoV-2 PCR assay of 180 clinical samples were 25.02 (first to third interquartile range, 24.81–25.13), 25.13 (first to third interquartile range, 25.01–25.26), and 25.26 (first to third interquartile range, 25.12–25.41) with nucleic acid extracted using the T-Prep24 system, TANBead system, and Qiagen system, respectively. Among the 180 clinical samples, 70 were positive with nucleic acid extracted using the T-prep24 system, and no discrepant results was identified when nucleic acid was extracted using the TANBead system. When the nucleic acid was extracted using the Qiagen system, two cases with indeterminate results were identified in 110 negative samples (Table 3).

**Figure 3:**
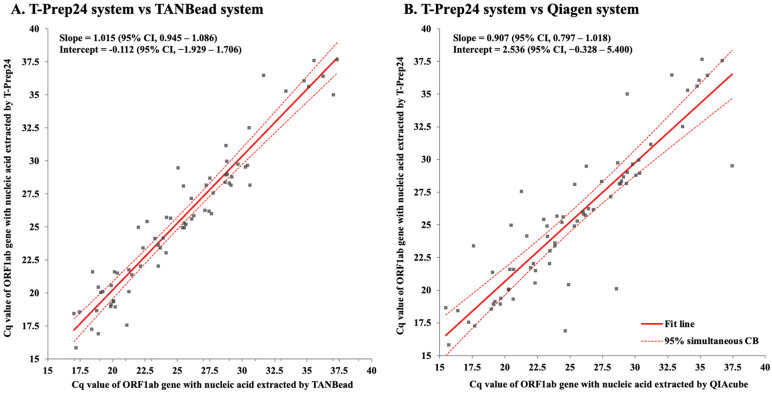
Comparison of Cq values of *ORF1ab* according to the extraction system. The correlation of Cq values of *ORF1ab* in SARS-CoV-2 PCR assays analyzed with Passing-Bablok regression showed minimal proportional bias between T-Prep24 and TANBead system, but there was substantial bias between T-Prep24 and Qiagen systems. (**A**) T-Prep24 system vs. TANBead system. (**B**) T-Prep24 system vs. Qiagen system.

The Cq values of *ORF1ab* of positive results with nucleic acid extracted by three systems were compared (Figure 3). The slope and intercept of equation between the results with nucleic acid extracted using the T-Prep24 system and the TANBead system were 1.015 and −0.112, respectively. The slope and intercept of equation between the results with nucleic acid extracted using the T-Prep24 system and the Qiagen system were 0.907 and 2.536, respectively.

## 4. Discussion

The applications of molecular diagnosis in clinical laboratories have been increasing in detection of bacterial or viral pathogens. During the COVID-19 pandemic, the gold standard for identifying patients with COVID-19 was PCR-based SARS-CoV-2 detection [8]. For the molecular genetic diagnosis, all steps, including specimen collection, nucleic acid extraction, and amplification, should be appropriately performed to obtain accurate results. In the first wave of rapidly increasing phase of COVID-19 pandemic in early 2020, one of the most important national goals to respond to urgent public threat was to expand capacity of SARS-CoV-2 PCR examinations. However, global supply shortage of nucleic acid equipment for nucleic acid extraction and amplification slowed down capacity expansion of laboratories in South Korea [9], and the need to develop domestic products of nucleic acid extraction equipment increased.

The T-prep24 system was developed using a new silica magnetic bead-based nucleic acid separation. The magnetic beads have a negative charge in their surface and selectively bind to extracted nucleic acid to wash out proteins and cellular debris. The adsorption of extracted nucleic acid by magnetic beads does not require centrifugation which might cause degradation of nucleic acids by generating shear forces [10]. In addition, this system requires shorter processing time, limited chemical use, and affordable costs compared with the conventional methods. Therefore, many commercially available nucleic acid extraction kits have been introduced with magnetic bead-based nucleic acid separation systems, including TANBead and Promega Magazorb, and they showed reliable extraction performance for clinical uses [11,12]. In the T-Prep24 system, four types of magnetic beads including 200 nm, 500 nm, 1000 nm, and 420 nm-diameter beads (Nanobrick, Gyonggido, Republic of Korea) with two different functional groups (-OH and -COOH) were evaluated with purity and extraction yield, and 410 nm -COOH magnetic beads was included in the BPDX-Viral DNA/RNA kit.

Accurate quantification of extracted nucleic acid is an important step in molecular diagnosis, and spectrophotometry, fluorometric assays, or capillary electrophoresis can be performed. The spectrophotometry measuring absorbance at 260 nm is a rapid method, but it is prone to overestimation in the presence of contaminants [13], and can be less sensitive at low concentrations. Fluorometric assays using dyes which selectively bind to DNA or RNA showed higher sensitivity and specificity [14]. In this study, the concentrations of extracted nucleic acid were quantified by both spectrophotometry and fluorimetry. The concentrations of extracted nucleic acid by spectrophotometry showed to be higher than those by fluorimetry regardless of the extraction system, which might be due to the contaminants. The nucleic acid quantification by fluorimetry was reported to be accurate when compared with the quantitative PCR assays, which should be considered as a gold standard for measuring nucleic acid quantity [15]. Therefore, fluorimetry should be adopted for the accurate quantification of nucleic acid.

By measuring the concentration of extracted nucleic acid by fluorescent method, the nucleic acid yields from the T-Prep24 system and TANBead systems were comparable, while the yield from the Qiagen system was approximately twice as high as that of T-Prep24. However, the A260/280 ratios of nucleic acids extracted by the T-Prep24 and TANBead systems were similar, likely reflecting the similarity in their magnetic bead-based extraction methods, while that extracted using the Qiagen system was much higher than that extracted using the T-Prep24 system. The A260/280 ratio is a measure of the purity of extracted nucleic acid, and a ratio below 1.8 is generally considered “pure” [16]. This finding might be related to the results of the SARS-CoV-2 PCR assays in this study. The PCR results were more reliable when using nucleic acids extracted by the T-Prep24 and TANBead systems, whereas two indeterminate results were observed with nucleic acids extracted by the Qiagen system, which were determined as negative results considering clinical findings. Furthermore, nucleic acids extracted using the T-Prep24 and TANBead systems yielded proportionally higher Cq values compared to those extracted using the Qiagen system, suggesting reduced sensitivity but increased specificity. This discrepancy may be attributed to the advantages of magnetic bead-based nucleic acid separation, which can more effectively reduce contaminants that interfere with amplification.

One of the limitations of this study is that only nasopharyngeal swabs were included in the evaluation. Further investigations with other specimens should be carried out to expand the clinical usefulness of this extraction system. In addition, the extraction efficacy of the system should be further evaluated using other respiratory RNA viruses as well as DNA viruses.

In conclusion, the performance of the T-Prep24 automated nucleic acid extraction system showed lower concentrations compared with two different nucleic acid extraction systems but showed a reliable concordance rate when compared with the results of real-time PCR for detecting SARS-CoV-2. This automated nucleic extraction kit could offer clinical utility in the detection of important respiratory pathogen.

## Figures and Tables

**Table 1:** Overview of the automated extraction platforms.

	T-Prep24 System	TANBead System	Qiagen System
Manufacturer	BioPark Diagnostics Inc. (Seoul, Republic of Korea)	Taiwan Advanced Nanotech Inc. (Taoyuan City, Taiwan)	Qiagen (Hilden, Germany)
Extractor	T-Prep24	SLA-E13200	QIAcube
Technique	Nucleic acid capture by magnetic bead	Nucleic acid capture by magnetic bead	Nucleic acid capture by silica gel membrane
Extraction kit	BPDX-Viral DNA/RNA Extraction Kit	TANBead Nucleic acid extraction kit	QIAamp DSP Viral RNA Mini Kit
Sample loading volume	200 μL		140 μL
Sample processing volume	50–1200 μL	50–1000 μL	
Elution volume	50–100 μL	70–100 μL	50 μL
Maximum throughput	24 samples/run	32 samples/run	12 samples/run
Running time	30 min	30–40 min	20–40 min
Cost—extractor	$20,000	$30,000	$40,000
Cost—extraction kit/test	$2–2.5/test	$3–4/test	$4–6/test

**Table 2:** Concentration and purity of extracted nucleic acid and results of PCR assay according to the extraction system.

	T-Prep24 System	TANBead System	Qiagen System
Concentration by Qubit, median (quartile range), ng/μL	0.685 (0.258–1.493)	0.985 (0.610–1.583) *	4.710 (3.783–5.810) *
Concentration by Nanodrop, median (quartile range), ng/μL	2.105 (0.539–8.841)	7.961 (6.274–10.359) *	83.275 (74.434–87.454) *
A260/A280 ratio	1.528 (1.273–1.825)	1.511 (1.412–1.611)	2.652 (2.594–2.708) *
SARS-CoV-2 PCR results			
Cq value of internal control, median (quartile range)	25.02 (24.81–25.13)	25.13 (25.01–25.26)	25.26 (25.12–25.41)
Positive	70	70	70
Cq value of *ORF1ab*, median (quartile range)	25.35 (21.42–28.76)	25.22 (20.54–28.79)	24.76 (20.82–29.07)
Cq value of *E* gene, median (quartile range)	24.21 (20.25–27.40)	22.85 (18.71–26.91)	22.86 (18.95–27.04)
Indeterminate	0	0	2
Negative	110	110	108

* *p* value < 0.001 compared with the value of T-prep24 system.

**Table 3:** Discrepant results.

Case Number	SARS-CoV-2 (T-Prep24)	SARS-CoV-2 (TANBead)	SARS-CoV-2 (QIAcube)
71	Negative	Negative	Indeterminate (38.17/-)
99	Negative	Negative	Indeterminate (38.52/38.72)

## Data Availability

The original contributions presented in this study are included in the article. Further inquiries can be directed to the corresponding authors.
